# From the Skin to the Brain: Pathophysiology of Colonization and Infection of External Ventricular Drain, a Prospective Observational Study

**DOI:** 10.1371/journal.pone.0142320

**Published:** 2015-11-10

**Authors:** Roman Mounier, David Lobo, Fabrice Cook, Mathieu Martin, Arie Attias, Bouziane Aït-Mamar, Inanna Gabriel, Olivier Bekaert, Jean Bardon, Biba Nebbad, Benoît Plaud, Gilles Dhonneur

**Affiliations:** 1 Department of Anesthesia and Surgical Intensive Care, Henri Mondor University Hospital of Paris, Paris XII school of medicine, Créteil, France; 2 Department of Neurosurgery, Henri Mondor University Hospital of Paris, Paris XII school of medicine, Créteil, France; 3 Department of Microbiology, Henri Mondor University Hospital of Paris, Paris XII school of medicine, Créteil, France; 4 Department of Anesthesiology and Surgical Intensive Care, Saint-Louis University Hospital of Paris, Paris VII school of medicine, Paris, France; Heinrich-Heine University, GERMANY

## Abstract

Ventriculostomy-related infection (VRI) is a serious complication of external ventricular drain (EVD) but its natural history is poorly studied. We prospectively tracked the bacteria pathways from skin towards ventricles to identify the infectious process resulting in ventriculostomy-related colonization (VRC), and VRI. We systematically sampled cerebrospinal fluid (CSF) on a daily basis and collected swabs from both the skin and stopcock every 3.0 days for microbiological analysis including in 101 neurosurgical patient. Risk factors for positive event defined as either VRC or VRI were recorded and related to our microbiological findings. A total of 1261 CSF samples, 473 skin swabs, and 450 stopcock swabs were collected. Skin site was more frequently colonized than stopcock (70 (60%) vs 34 (29%), p = 0.023), and earlier (14 ±1.4 vs 24 ±1.5 days, p<0.0001). Sixty-one (52%) and 32 (27%) skin and stopcock sites were colonized with commensal bacteria, 1 (1%) and 1 (1%) with pathogens, 8 (7%) and 1 (1%) with combined pathogens and commensal bacteria, respectively. Sixteen positive events were diagnosed; a cutaneous origin was identified in 69% of cases. The presence of a pathogen at skin site (6/16 vs 4/85, OR: 11.8, [2.5–56.8], p = 0.002) and CSF leakage (7/16 vs 6/85, OR 10 [2.4–41.2], p = 0.001)) were the two independent significant risk factors statistically linked to positive events occurrence. Our results suggest that VRC and VRI mainly results from an extra-luminal progression of pathogens initially colonizing the skin site where CSF leaks.

## Introduction

External drainage of CSF is commonly used in neurosurgery. A major complication of EVD is VRI [1,2]. Reported incidences of drain-related meningitis ranges from 2 to 22% for EVD [1–6] and a meta-analysis reported a cumulative rate of positive CSF cultures of 8.8% per patient or 8.1% per EVD [7]. Factors associated with increased risk of infection are intraventricular or subarachnoid hemorrhage, craniotomy, systemic infection, cranial fracture with CSF leakage and EVD irrigation [1,2,7].

Early detection of a VRI is essential for a successful treatment. Clinical and biological patterns of VRI are non-specific [8,9], CSF parameters have not been shown to be predictive for an infection [2,7,8,10–12] and microbiological analysis of CSF, despite being highly specific for VRI, are not sensitive [13] and requires time, thus most authors recommend a dynamic analysis of CSF [6,7]. When and how often CSF should be sampled and investigated in patients with EVD is controversial [2,6,14–19]. Furthermore it remains unclear whether EVD manipulation increases the risk of infection [4,20–23].

VRI results from previous colonization. Different sources of CSF-drain colonization are possible: during insertion, during disconnection or manipulation (intraluminal route), or colonization of the drain at the insertion site (extraluminal route). Hematogenous seeding is another potential route, probably less common. For intravenous device, skin appears to be the primary source of device-related bacteremia for short-term catheters (<8 days) [7,24]. For VRI, gram-positive cocci consistent with skin flora represents the majority of isolates from CSF infection in most of the studies [7].

The purpose of this study was to identify the major route of EVD-colonization. The second objective was to assess the risk factors for VRI. Finally we aim to evaluate whether VRC with pathogenic bacteria is or not closely related to VRI.

## Methods

### Patients

In this prospective study from September 2009 through February 2011, all patients older than 18, hospitalized in the neurosurgical intensive care unit (ICU) of a tertiary care university center and who received EVD for at least 48 hours, were included. Exclusion criteria were EVD-indwelling time <48 hours, infection of an internal CSF shunt, intracranial infection, skull fracture, aseptic meningitis, and meningitis which developed within the 48^th^ hour after the EVD placement or ≥5 days after the EVD was removal.

The Institutional Ethical Committee approved the study (Comité de Protection des Personnes, Paris IX). Written consent was obtained initially from the relatives of the patient, or during the hospital stay for those that survived

### Data collection

We evaluated patients on a daily basis and prospectively collected the following data, until VRI or EVD removal: patient characteristics; underlying neurosurgical condition; reason for EVD placement; date of EVD insertion and EVD removal; prophylactic antibiotic administration; continuous CSF diversion or not; the drainage volume, and all events that resulted in manipulation of the system (including CSF sampling and irrigation, disconnections of the system, CSF leakage); stopcock manipulation; daily clinical signs and the presence of other infections (organisms, antimicrobial susceptibility, treatment provided); blood leukocyte count; RBC; blood glucose; serum chemistry; daily CSF laboratory parameters, CSF gram stain and culture, culture of EVD tip, antimicrobial therapy for treatment of other infection sites (expressed as: ATB during EVD drainage); treatment outcome. For more details on the recorded data collection, please refer to Supporting Information File (S1 File).

### Catheter insertion, maintenance and manipulation and removal procedures

#### Catheter insertion

During the study period, Bactiseal^®^ EVDs (Bactiseal, Codman, Johnson&Johnson, Wokingham, UK) were exclusively used. The bactiseal^®^ EVD diffuses a combination of antibiotics (clindamycin and rifampicin), so that both the inner lumen and the exterior catheter wall are coated up to 28 days with antibiotic concentrations that protect against bacterial colonization [25]. EVDs are inserted under aseptic condition in the operating room. Prior to EVD insertion, the patient’s skull was shaved and skin prepared with standard sterile techniques (*povidone-iodine solution*). Our institutional policy does not recommend antimicrobial prophylaxis immediately before EVD placement or CSF drainage. However antibiotic prophylaxis can be injected mainly depending upon local conditions evaluated by the surgeon. For EVD placement in surgical environment, the catheter is introduced through a precoronal burr hole into the ventricle. CSF is obtained through the catheter and sent for analysis. Then, the catheter is subcutaneously tunneled (>5 cm) and sutured [26]. Finally an occlusive dressing is placed to cover the 2 cutaneous incisions.

#### Catheter maintenance

Hand hygiene is a common standard protocol in the ICU and particularly while maintaining and manipulation EVD system. It includes at least 2 hands washing with hydro-alcoholic solution, one just before and one just after EVD handling.

Our procedures of EVD maintenance imposes EVD insertion sites are cleaned (*povidone-iodine procedure*) at bedside and redressed every 48 hours, unless if soiled, as defined in our institutional policy. For this purpose, the ICU nurse wearing hair cap and a face mask removes the covering dressing with sterile gloves put after standard hands hygiene. The sites are inspected for CSF leaks or local skin infection signs. If hair grows thus preventing the occlusive dressing to adhere, the nurse shave the surrounding would skin area. The nurse then removes the first pair of gloves and standard hands hygiene is performed for a second time, before putting again a pair of sterile gloves. A new sterile gauze dressing is applied to the site, and benzoin is used to hold the tape???. Our policies recommend the dressing is tightly occlusive. After application of a covering sterile dressing, the system remained entirely closed.

#### Catheter manipulation

A written protocol is systematically followed for catheter manipulations. The Bactiseal^®^ catheter is connected to a Codman^®^ EDS 3^™^ CSF External Drainage System with three 3-way stopcocks dedicated to 1-the “patient” line, 2- the “system” and 3- the “drip chamber”. The later is connected to a closed CSF collection bag (Johnson&Johnson, Wokingham, UK). The patient’s line stopcock is never used, except in case of obstruction, for fibrinolytic irrigation of the EVD system. The “system” stopcock is only used for CSF withdrawal procedure.

Assessment of the drainage system is done every 3 hours, which includes inspecting the EVD from the insertion site along the entire drainage system, checking for cracks or fluid leakage. The sampling of the CSF is performed daily via the drip chamber stopcock following a standardized aseptic technique including: hair covering cap, face mask, hand hygiene, sterile gloves sterile field, and disinfection of the connecting sites using chlorhexidine-ethanol solution. Only ICU staffs were involved with the sampling procedure. The external collecting bag of the drainage system are changed on a daily basis using sterile gloves and a mask.

#### Catheter removal

EVDs is removed after CSF sampling under aseptic conditions in the ICU and the catheter is sent to the laboratory of microbiology.

### Bacteriological specimens and investigation

Specimens for microbiological study were obtained from the second day and at the time of each dressing changes (every 2 days) until EVD withdrawal or VRI. They included swabs (Eswab; Copan, Brescia, Italy) from the skin surrounding the catheter entry site (an area of 4 cm^2^) and from the drip chamber stopcock, obtained throughout the catheterization period. Moistened swabs were used to obtain specimens from the skin. These specimens were obtained by rolling a sterile-tipped applicator over the skin. Two sets of back-and-forth strokes were performed at right angles to each other. Inner stopcock samples were taken by rubbing (360°) the interior surface of the stopcock with a sterile applicator. Each applicator was placed in transport medium and sent to the laboratory.

CSF samples were sent to the laboratory for direct examination, Gram stain, and culture. The tip was sonicated then vortexed, and subsequently streaked on blood agar plates. Swab samples were streaked on blood agar plates (Tryptone soja agar (TSA), Columbia, and Drigalski agar) and incubated at 35°C. CSF sample were sent to the laboratory for direct examination, Gram stain, and culture. Culture media included 5% sheep blood agar, chocolate agar (in 5% CO_2_), TSA, MacConkey agar and Schaedler broth. Inoculated media were incubated at 35–37°C, for 5 days. Bacterias were identified by means of standard biochemical test. Susceptibility testing was performed by the agar dilution replicate plating method. All cultures were incubated under aerobic and anaerobic conditions.

All strains were conserved at the microbiological laboratory. The strain relatedness was assessed by determination of species and antibiotyping [27].

### Main outcome parameters

Bacterias were divided into 2 groups: pathogenic and skin commensal, (such as *Propionibacterium*, *Corynebacterium* and Coagulase-negative *Staphylococcus* (CoNS)).

VRI was defined as a positive CSF culture obtained from the ventricular catheter [2,3,14,17–19,28–35], except for commensals, where the same strain had to be isolated in two or more CSF samples [36].

Ventriculostomy-related colonization (VRC) was defined as a positive culture of catheter tip with no positive culture of CSF. For pathogens, any number of colony-forming units was considered positive; for commensals, a cut-off of ≥ 15 colony-forming units was applied [37].

VRC and VRI were defined as positive event.

The presumed source of VRI/VRC included the following possibilities:

- Hematogenous seeding: the same strain was to be isolated from cultures of the catheter tip or CSF and from blood cultures and/or from a known distant source of infection, in conjunction with both negative stopcock and skin culture.
- Skin: the same strain has to be first isolated from cultures of skin, and then from CSF or catheter tip. Stopcock cultures could be either negative or positive for different strains.
- Stopcock: the same strain has to be first isolated from cultures of the drip chamber stopcock, and then from CSF or catheter tip. Skin cultures could be either negative or positive for different strains.
- Unknown: the strain never isolated before, or present on the stopcock and the skin before the positive event occurred.

### Statistical analysis

This trial was not sized preemptively. We decided including all consecutive EVD patients admitted in our surgical intensive care unit over the study period. Qualitative data are expressed as number and percentage, and quantitative data as median and interquartile range (ICR). Categorical data were compared with Fisher Exact test. Quantitative data were compared with Mann-Whitney U test. Positive event associated factors identified by univariate analysis (with p<0.2 threshold) were subsequently used to conduct a multivariate analysis with logistic regression, using a stepwise backward elimination method. Coefficients were estimated with maximal likelihood method. Skin and stopcock colonization rates were estimated by Kaplan-Meier method and compared with Log-rank test. Finally, we calculated *a post hoc* power based on our observed proportions of external pathway factors that were our main hypothesis to explain infection. For more details on *post-hoc* power calculation, please refer to Supporting Statistical Information File (S2 File).

P values were two-sided, and considered to indicate statistical significance under 0.05. Odds-ratios were expressed with 95% confidence interval.

Analysis were performed using IBM^®^ SPSS statistics 20.0^®^ software.

## Results

### Characteristics of the studied population

**Fig 1:**
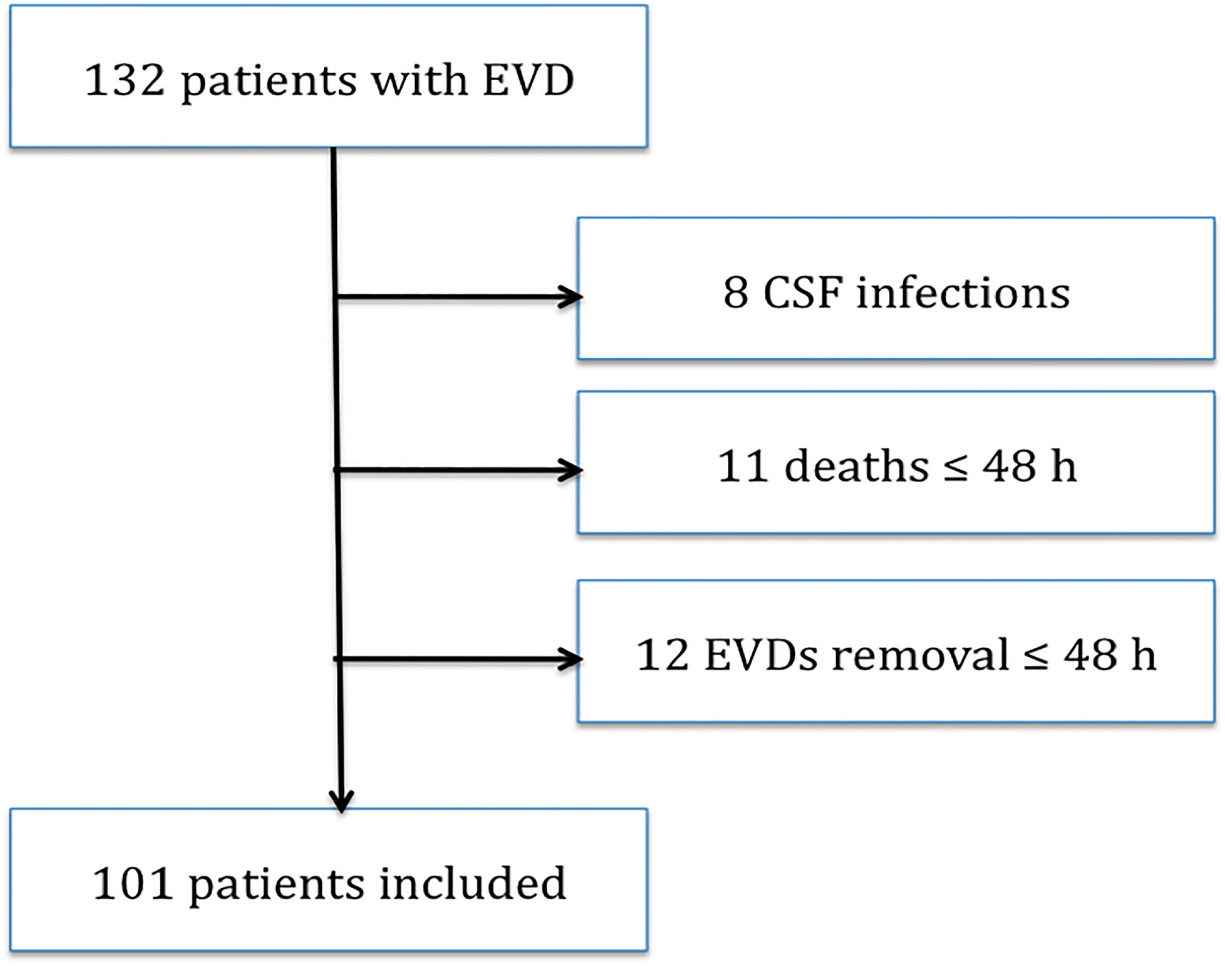
Flow Chart of the patients. 132 patients older than 18 year-old received external ventricular drainage for at least 48 hours. From September 2009 to February 2011, 101 patients were included.

During the study period 101 patients that underwent placement of 116 EVDs were included (Fig 1). Population and EVD placement characteristics are shown in Table 1.

**Table 1:** Baseline characteristics of patients.

Caracteristic	n = 101
Age (years)	52 [41–62]
Male sex	50 (49,5)
SAPS 2	28 [15–42]
ASA score	
1	46 (45,5)
2	46 (45,5)
3	9 (8,9)
Causes of ICU admission	
aSAH	67 (66,3)
Hemorragic stroke	12 (11,9)
IC tumor	6 (5,9)
TBI	4 (4)
Other	12 (11,9)
Initial GCS score	11 [6–14]
Fisher grade	
2	2 (2,8)
3	7 (9,7)
4	63 (87,5)
WFNS grade	
≤ 3	40 (56,3)
> 3	31 (43,7)
Immunodeficiency	4 (4)
Intraventricular hemorrage	79 (78,2)
Indication of drainage	
Hydrocephalus	101 (100
Number of drains per patient	
1	86 (85,1)
2	14 (13,9)
3	1 (1)
Length of catheterization (days)	13 [9–18]
Lengh of ICU stay (days)	18 [12–24]
ICU death	23 (22,8)

NOTE. Data are shown as median [25th - 75th percentil] or n(%). SAPS 2, simplified acute physiology score 2; ASA score, American Society of Anesthesiologists score; ICU, intensive care unit; GCS: Glagow coma scale; aSAH, aneurysmal subarachnoid hemorrhage; IC tumor, intracranial tumor; TBI, traumatic brain injury; WFNS grade, World Federation of Neurosurgical Societies grade

### Positive events incidence

Sixteen patients (16%) developed a positive event representing a positive event incidence density of 10.9 per 1000 days of catheterization. Ten patients (10%) developed a VRI, representing an incidence rate of infection of 6.8 per 1000 days of catheterization. As analyzed with Kaplan-Meier curve in order to take into account censored data, the mean (SD) length of catheterization before VRI was 32 (2.2) days. Eighty percent of VRIs (n = 8) were caused by a pathogen (Table 2). The mortality rate of VRI was 20% (2 of 10).

**Table 2:** Microbiological findings for positive events.

		Age (years)	Gender	ICU death	Tip culture	Pathogen	Consistent positive skin swab^a^	Consistent positive stopcock swab^a^	Unknown pathway	Skin colonisation by High pathogen
VRI	1	60	F	Yes	*E*.*Cloacae*	*E*.*cloacae*	Yes		.	Yes
	2	74	F		Sterile	*E*.*faecalis*	Yes		.	Yes
	3	52	F		Sterile	*S*. *aureus*	Yes		.	Yes
	4	58	M		*S*. *epidermidis*	*S*. *epidermidis*		Yes	.	
	5	60	F		-	*E*.*faecalis*			Yes	
	6	24	M		*S*. *epidermidis*	*E*.*faecalis*	Yes		.	Yes
	7	76	M	Yes	Sterile	*Streptococcus spp*.			Yes	
	8	30	M		Sterile	*S*. *epidermidis*	Yes		.	
	9	50	M		*K*. *pneumoniae*	*K*. *pneumoniae*			Yes	
	10	28	M		*E*.*coli*	*E*.*coli*	Yes		.	Yes
VRC	1	59	M		*S*. *epidermidis*	*S*. *epidermidis*	Yes		.	
	2	61	F		*S*. *epidermidis*	*S*. *epidermidis*	Yes		.	
	3	34	M		*E*.*cloacae*	*E*.*cloacae*	Yes		.	Yes
	4	46	M		*S*. *epidermidis*	*S*. *epidermidis*	Yes		.	
	5	54	F		*S*. *epidermidis*	*S*. *epidermidis*	Yes		.	
	6	30	M		*S*. *epidermidis*	*S*. *epidermidis*			Yes	

**NOTE**.

^a^. Before positive évent.

VRI, ventriculostomy related infection; VRC, ventriculostomy related colonization; F, female; M. male; ICU, intensive care unit.

Ninety-seven tips were analyzed with positive culture for 10 tips (10%). Among these, 6 were classified as VRC, representing a colonization incidence density of 4.1 per 1000 days of catheterization.

For the 10 VRIs, 9 had the tip analyzed, with concordant strains in 4 positive cultures. For one VRI, the strain found on the tip was different from the strain found in both the CSF and swabs (Table 2).

One hundred-one cultures were performed at the moment of the primary catheter insertion. Two of these cultures were positive, but where considered to be contaminants.

### Cutaneous and stopcock colonization

A median [ICR] of 4 [3–6] skin and 4 [2–6] stopcock swabs were obtained per patient. Among the 473 skin swabs, 164 (34.7%) cultures were positive and among the 450 stopcock swabs, 46 (10%) cultures were positive. (Table 3).

**Table 3:** EVDs microbiological data (n = 117).

***Overall***	
Length of drain (days)	1469
Skin swab samples	473
Stopcock swab samples	450
CSF cultures	1261
***Per EVD*** ^a^	
LOC (days)	12 [8–16]
Skin swab samples	4 [2–5]
Frequency of skin swab samples	3 [2.6–4]
Positive skin swab samples	1 [0–2]
Skin swab cultures	
Sterile	47 (40.2)
Low pathogen	60 (51.3)
Pathogen	1 (0.9)
Both	9 (7.7)
Stopcock swab samples	3 [2–5]
Frequency of stopcock swab samples	3.2 [2.7–4]
Positive stopcock swab samples	0 [0–1]
Stopcock swab cultures	
Sterile	83 (70.9)
Low pathogen	32 (27.4)
Pathogen	1 (0.9)
Both	1 (0.9)
EVD remaining sterile at both sites	39 (33.3)
CSF cultures	10 [6–14]
Tip cultures	
Sterile	87 (89.7)
Low pathogen	9 (9.3)
High pathogen	1 (1)

^a^ Data are shown as median [25th - 75th quartils] or n (%)

CSF, cerebro-spinal fluid; LOC, length of catheterization; EVD, external ventricular drain.

**Fig 2:**
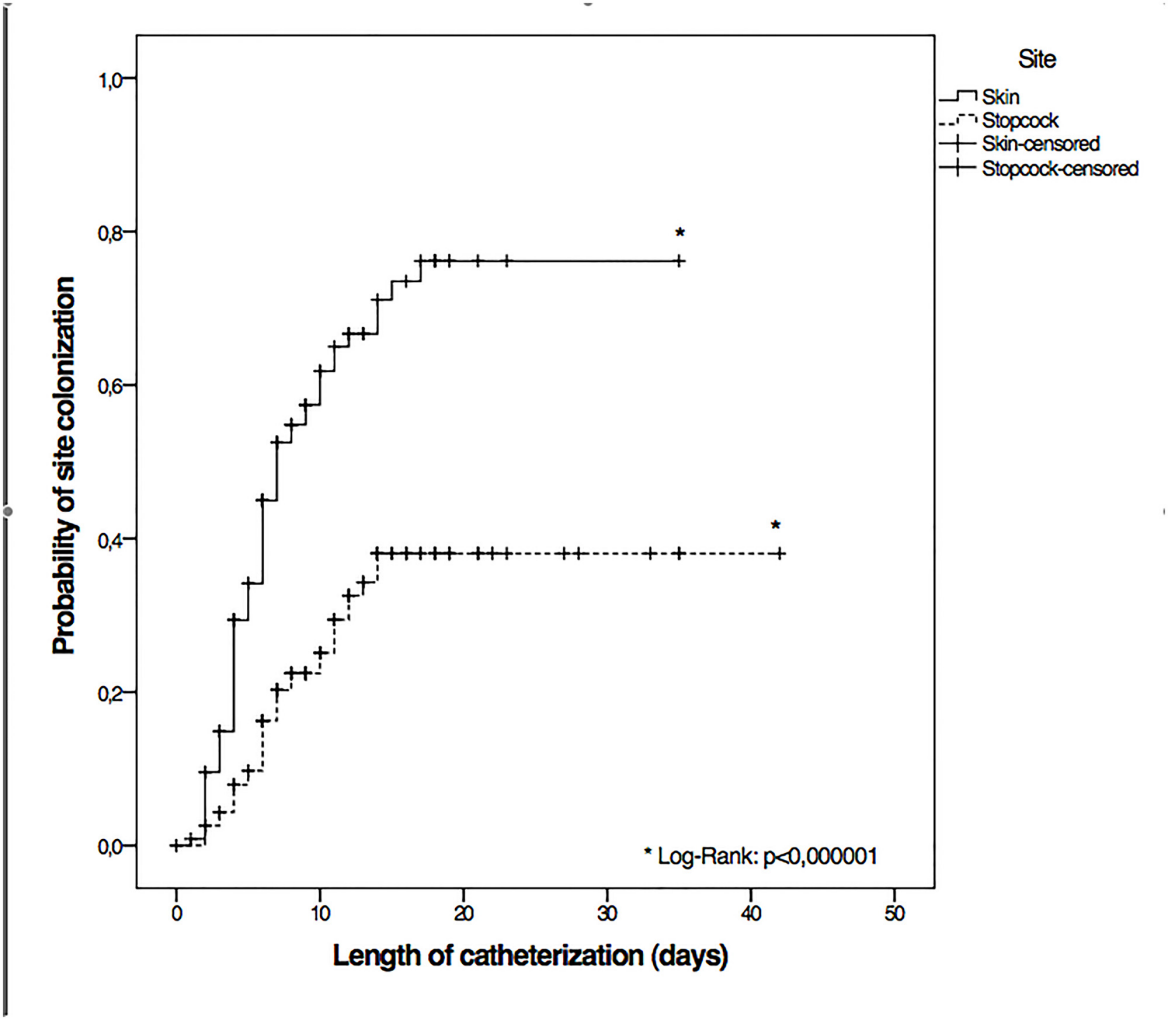
Kaplan-Meier analysis for probability of skin and stopcock colonization.

Skin colonization was significantly more frequent than stopcock colonization (60% vs 29%, p<0.0001), with a mean (SD) time until swab positivity of 14.0 (1.4) vs 24.0 (1.5) days, respectively (log rank p<0.0001) (Fig 2).

CoNS accounted for almost all bacteria isolated from skin and stopcock colonization.

When colonized, surveillance culture of the site inconsistently found the microorganism. Only 39 (33%) EVDs placed in 25 patients were associated with negative surveillance cultures of both the stopcock and skin. The incidence of positive events in these patients was 2/25 (8%) (1 VRI and 1 VRC), versus 14/76 (18%) when there was at least one positive sample (p = 0.34).

### Sources of strains found in Positive Events

Skin was identified as the major source of positive events (Table 2): 60% of VRI and 83% of VRC were associated with previous skin colonization related to the same strain. No positive events presented the same strain on both skin culture and stopcock culture. In 4 positive events, no source of contamination was identified: there was no prior bacteremia, pneumonia or urinary tract infection. The skin source for a positive event was significantly more frequent than the stopcock source (p = 0.006), but not significantly different than from unknown origin (p = 0.12).

The median positivity time of the surveillance skin culture preceded VRI of 1.5 [0–9] days, and the median time elpasing between positivity of stopcock culture and VRI was 5 days.

Colonization with pathogen was significantly more frequently associated with positive event than colonization with CoNS (6/10 patients (60%) *vs* 5/67 patients (7%), respectively, p<0.001).

### Risk factors for Positive Events

Patient’s characteristics and neurosurgical factors were not associated with the risk of developing positive events (Table 4). The length of catheterization tended to be higher in patients with positive events (16 [13–20] vs 13 [9–17] days, p = 0.065).

**Table 4:** Univariate and multivariate analysis of risk factors associated with positive events.

	Positive event (VRI or VRC)		Univariate analysis		Multivariate analysis	
	No (n = 85)	Yes (n = 16)	OR [95% CI]	p-value	OR [95% CI]	p-value
Age	52 [41–63]	53 [32–60]	-	0.714	-	-
Male sex	40 (47.1)	10 (62.5)	1.9 [0.6–5.6]	0.288	-	-
Cause of ICU admission						
aSHA	55 (64.7)	12 (75.0)	-	0.574	-	-
Hemorragic stroke	11(12.9)	1 (6.2)	-		-	-
IC tumor	6 (7.1)	0 (0)	-		-	-
TBI	4 (4.7)	0 (0)	-		-	-
AVM	1(1.2)	1 (6.2)	-		-	-
Other	8 (9.4)	2 (12.5)	-		-	-
Immunodeficiency	4 (4.7)	0 (0)	-	1	-	-
Recent neurosurgery	5 (5.9)	0 (0)	-	1	-	-
IVH	65 (76.5)	14 (87.5)	2.2 [0.5–10.3]	0.512	-	-
ATB during EVD placement	12 (14.5)	1 (6.2)	0.4 [0.05–3.3]	1	-	-
ATB during EVD drainage	21 (24.7)	6 (37.5)	1.8 [0.6–5.6]	0.357	-	-
EVD placement by resident	35 (42.7)	6 (37.5)	0.8 [0.3–2.4]	0.787	-	-
Emergency EVD placement	84 (98.8)	15 (93.8)	0.2 [0–3]	0.293	-	-
LOC (days)	13 [9–17]	16 [13–20]	-	0.065	1 [0.9–1.1]	0.38
EVD exchange	11 (12.9)	3 (18.8)	1.6 [0.4–6.3]	0.692	-	-
Drainage lock	36 (42.4)	8 (50.0)	1.4 [0.5–4]	0.594	-	-
EVD disconnection	10 (11.8)	2 (12.5)	1.1 [0.2–5.4]	1	-	-
Proximal sampling of CSF	10 (11.8)	3 (18.8)	1.7 [0.4–7.1]	0.428	-	-
CSF leakage	6 (7.1)	7 (43.8)	10.2 [2.8–37.2]	<0.001	10 [2.4–41.2]	0.001
EVD irrigation	9 (10.6)	1 (6.2)	0.6 [0.1–4.8]	1	-	-
Other systemic infection	20 (23.5)	6 (37.5)	1.9 [0.6–6.0]	0.348	-	-
Skin swab culture						
Sterile	30 (35.3)	3 (18.8)	-	<0.01	-	-
Low pathogen	51 (60.0)	7 (43.8)	-		-	-
Pathogen	0 (0)	1 (6.2)	-		-	-
Both	4 (4.7)	5 (31.2)	-		-	-
Skin colonisation by pathogen	4 (4.7)	6 (37.5)	12.1 [2.9–50.5]	<0.001	11.8 [2.5–56.8]	0.002
Stopcock swab culture						
Sterile	56 (65.9)	11 (68.8)	-	1	-	-
Low pathogen	27 (31.8)	5 (31.2)	-		-	-
Pathogen	1 (1.2)	0 (0)	-		-	-
Both	1 (1.2)	0 (0)	-		-	-
Stopcock colonisation by pathogen	2 (2.4)	0 (0)	-	1	-	-
Number of drains per patient						
1	73 (85.9)	13 (81.2)	-	0.738	-	-
2	11 (12.9)	3 (18.8)	-		-	-
3	1 (1.2)	0 (0)	-		-	-

NOTE. Data are no.(%) of patients, unless otherwise indicated. Categorical data were compared with Fisher Exact test. Quantitative data were compared with Mann-Whitney U test. Multivariate analysis was performed using logistic regression. VRI, Ventriculostomy Related Infection; VRC, Ventriculostomy Related Colonization; OR, Odds Ratio; CI, Confidence Interval; ICU, Intensive Care Unit, aSAH, aneurysmal Subarachnoid Hemorrhage; IC tumor, Intracranial Tumor; TBI, Traumatic Brain Injury; AVM, Arterio-Venous Malformation; IVH, Intraventricular Hemorrhage; ATB, Antibiotic Administration; EVD, External Ventricular Drain; LOC, Length Of Catheterization; CSF, Cerebro-Spinal Fluid.

Results of univariate analysis are shown in Table 4. In a subsequent logistic regression, CSF leakage and the presence of a pathogen at the insertion site remained independent risk factors (OR = 10 [2.4–41.2], p = 0.001; and OR = 11.8, [2.5–56.8], p = 0.002; respectively) of positive event. Interruption of the closed system did not appear to increase the risk of positive event.

### Conservative diagnosis

With surveillance swabs performed independently from suspicion of infection, the sensibility, specificity, positive and negative predictive value for developing a positive event was 81%, 35%, 19%, and 91% respectively. The positive and negative likelihood ratios were then 1.26 and 0.53. The sensibility, specificity, positive and negative predictive values for developing a positive event when skin swab had a positive culture for pathogen was 38%, 95%, 60%, and 89%, respectively.

## Discussion

We prospectively studied the fate of 117 EVD placed in 101 patients with the objective to identifying the major route of colonization and infection of CSF drainage system. Ten patients developed VRI and 6 VRC. Skin colonization was the major route of both VRI and VRC. Skin colonization occurred more frequently and earlier than that of stopcock. Common skin flora was the major colonizing microorganism. Less frequently, some pathogens could colonize the skin. A logistic regression found that CSF leakage and the presence of a pathogen at insertion site were significant risk factors for both VRC and VRI.

Pathophysiology of EVD colonization is unclear since there are no studies assessing directly how colonization occurs. In this study, we consider colonization of EVD and VRI as a positive event. Thus, analyzing the natural history of VRI requires studying colonization as well [37].

Many studies, mainly retrospective, analyzed risk factors of VRI. These factors can be categorized into 3 groups: patient’s characteristics and neurosurgical factors; events breaking the integrity of a closed system; and environmental influences [1].

Regarding the first group of VRI risk factors, none of them was associated with a risk of developing a positive event in the cohort we have followed. Our observation is consistent with findings previously reported in the literature [2,7,16,18,21]. However, some authors reported an association between VRI and either intra-ventricular hemorrhage caused by SAH [2,3,7], insertion of the catheter in emergency situation [7], neurosurgical procedure associated with EVD placement [7,18] or the surgeon’s experience. We did not find such association, which might be due to a high prevalence of these risk factors the population we have studied. Indeed, 66% of our patients had an SAH, 78% suffered from intra-ventricular bleeding, and EVD was placed in emergency situation in 97% of case.

It seems that factors related to a break that occurs in a closed, sterile CSF drainage system were more constantly highlighted by studies, especially catheter manipulation. Irrigation and CSF leaks at insertion site have consistently been associated with increased CSF infection rates [1,2,7,15]. In our cohort, irrigation was rarely used; therefore it was not statistically associated with an increased risk of infection (Table 4). Bogdahn et al. found 13% of VRI when there was a CSF leak at catheter insertion site as compared with 1.6% when there was no leaks [15]. Lyke and al. reported a significant association between CSF leakage around the EVD and the development of CSF infection [1]. In our study, CSF leakage at skin insertion was an independent factor for development of positive event (OR = 10 [2.4–41.2], p = 0.001). In contrast, drainage system leaks and disconnections have rarely been associated with increased infection rates [1,2,7]. Accordingly, we found that planed or unplanned disconnection of EVD system did not increased the risk of developing positive events.

All these results and observation plead for an extraluminal migration of the microorganism resulting in CSF infection. Skin colonization at insertion site (Fig 2) occurred rapidly, and the dominant specie was CoNS (51% of EVDs). Manipulated stopcocks, despite a daily use, were less frequently colonized (overall colonized EVDs, 29%). Moreover, among our positive events, 11 (69%) were compatible with colonization from the skin at the entry site along the external surface of the catheter. Hoefnagel et al., reported that the mean (SD) number of CSF sampling was significantly higher in patients with a CSF infection [4.0 (3.7) vs 1.4(1.8); p<0.001] but in their cohort 23% of patients developed VRI, which represent a very high infection rate possibly associated with the fact that CSF sample were collected via the proximal stopcock [21]. Despite a more intensive use of distal stopcock (daily), our incidence rate of infection was lower than most of the reported incidences [2,7,20–22,37]. Length of catheterization was also frequently reported as risk factor of VRI [1,7,21]. Examination by Lozier et al., of the raw data of Winfield et al., suggested a gradual increase of the daily infection rate, peak between day 9 and day 11[7]. Studying central venous catheter (CVC), Raad et al. suggested that colonization originating from the skin were predominant in short-term CVCs, while that originating from the hub were predominant in long-term catheterizations [38]. Our median [IQR] duration of catheterization of 13 days [9–18] might be too short to highlight the role of stopcock colonization. However, the length of catheterization rarely exceeds 10 days in most studies [1,7,20,21,37] thus reducing the influence of the endoluminal route (through the stopcock) as a major element of the natural history of VRI development.

As might be expected with the use of percutaneous catheters (23), gram-positive infections (mainly *Staphylococcus*) traditionally have been predominant [1–4,7,16,20,37]. In our cohort, CoNS was commonly found on the skin and *S*.*epidermidis* accounted for the majority of VRC (5 of 6 VRCs), but this bacterial specie was under represented in case of VRI (2 of 10 VRIs). Inversely, when a pathogen grew on the skin, the risk of its association to a VRI was very high (OR = 11.8, [2.5–56.8], p = 0.002).

Few studies have clearly distinguished colonization from infection. Shade et al., reported a bacterial colonization of the drainage catheter in 8 patients (4%)[20]. Among these, 6 were caused by gram-positive bacteria, of which 3 were CoNS. For their 22 VRIs, 18 (82%) were caused by a gram-positive bacilli, of which only 8 CoNS; *S*.*aureus* and *E*.*faecalis* accounted for the remaining. Hetem et al., studying retrospectively the relationship between bacterial colonization of EVD and secondary meningitis, reported CoNS, *S*.*aureus*, and gram-negative bacilli in 49%, 16% and 12% of cultured tips, respectively [37]. Secondary meningitis occurred in 74%, 65%, and 48% of cases when tips were colonized by *S*.*aureus*, gram-negative organisms, and CoNS, respectively. *S*.*aureus* cultured from the tip was found as a significant risk factor associated with secondary meningitis [37]. In our cohort, it was the pathogenic characteristic of the bacteria which was significantly associated with the risk of infection. We then hypothesized that the virulence of a pathogen, once colonizing the skin, leads to a faster invasion, colonization and finally infection than a commensal would. This hypothesis could explain why, in the study of Hetem et al., almost every secondary meningitis (69%) developing 2 days or more after EVD removal (with positive culture of the tip) were caused by CoNS.

Regarding the diagnostic value of systematic skin swab series, we found low likelihood ratios, resulting in a poor improvement of post-test probability. Thus, systematic skin swab series for all patients cannot be recommended to assess infectious complications of EVD. However, we did not test the diagnostic value of a single skin swab in clinical condition of suspected infection.

Some limitations of our study need to be discussed. First, isolates of *S*.*epidermidis* were not studied by a method of genotypic analysis. Although it has been reported that species determination associated to antibiotype correlation allowed the assessment of strain relatedness among organisms within the same species with a high likelihood [27], it is possible that *S*.*epidermidis* were in fact genetically different. Second, Bactiseal EVD ^®^ were impregnated with antibiotics on all catheter (inner and outer) surfaces [39], therefore this EVD has not influenced one route over another. Interestingly, our positive events were caused by susceptible strains to these antibiotics. Third, despite more than 100 patients and according to the incidence rate of infection reported in the literature, we observed few infectious events. In the condition of our study, the frequency of skin or stopcock swab colonization was low, and a part of positive events of unknown origin would have been explained if this frequency was higher. Our study was not sized preemptively and the sample size of the study is possibly too small. However the power analysis we performed *a post hoc* evidenced a power exceeding 90% with our samples sizes. Moreover, we computed robust correlations. When studying a rare disease, researchers may use a retrospective design to increase the number of events, but such design is associated with a specific bias procession. Conducting a multicenter study would have certainly increased the number of patients with positive event, thus reinforcing the strength of the findings. However, standardization among centers of EVD handling policies would have been very difficult. We deliberately opted for single center study with very strict and uniform EVD handling both policies and procedures to limit methodological bias, and better respond to our pathophysiologic hypothesis. Although the small size sample of the study is objectionable, our results allow us to propose a reasonable hypothesis on the preferred route of colonization and infection of EVD. Other studies are needed, especially to assess the likelihood ratio according to the clinical context.

## Conclusion

We aimed to identify the dominant route of EVD-colonization, to assess the risk factors for VRI and to evaluate whether VRC with pathogenic bacteria is or not closely related to VRI. Our results suggest that VRC and VRI mainly results from an extra-luminal progression of pathogens initially colonizing the skin site where CSF leaks.

## Supporting Information

S1 File Supporting Information File: DataBase. (CSV)

S2 File Supporting Statistical Information File: Statistical Data Set. (DOCX)
