# Supplementary material for: From the Skin to the Brain: Pathophysiology of Colonization and Infection of External Ventricular Drain, a Prospective Observational Study
Source: PLoS One. 2015 Nov 10;10(11):e0142320. doi: 10.1371/journal.pone.0142320 (PMC4640851; doi:10.1371/journal.pone.0142320)
Supplement: S2 File — (DOCX) [file pone.0142320.s002.docx]

**Annex : full statistical data set**

**Power calculation with R**

*[1] "Power calculation for leak"*

*difference of proportion power calculation for binomial distribution (arcsine transformation)*

*h = 0.9050274*

*n1 = 16*

*n2 = 85*

*sig.level = 0.05*

*power = 0.9132509*

*alternative = two.sided*

*NOTE: different sample sizes*

*[1] "Power calculation for hight pathogen at the skin site"*

*difference of proportion power calculation for binomial distribution (arcsine transformation)*

*h = 1.335094*

*n1 = 16*

*n2 = 85*

*sig.level = 0.05*

*power = 0.9983546*

*alternative = two.sided*

*NOTE: different sample sizes*

**Logistic regression models with interaction terms :**

***Model with 2 way interaction term between LOC and CSF leak***

| **Variables dans l'équation** | | | | | | | | | |
| --- | --- | --- | --- | --- | --- | --- | --- | --- | --- |
|  | | A | E.S. | Wald | ddl | Sig. | Exp(B) | IC pour Exp(B) 95% | |
|  |  |  |  |  |  |  |  | Inférieur | Supérieur |
| Etape 1^a^ | Fuite_LCR_01(1) | 1,298 | 2,190 | ,351 | 1 | ,553 | 3,663 | ,050 | 267,760 |
|  | Duree_KT_tot | ,053 | ,039 | 1,844 | 1 | ,174 | 1,055 | ,977 | 1,138 |
|  | Duree_KT_tot by Fuite_LCR_01(1) | ,068 | ,136 | ,251 | 1 | ,617 | 1,071 | ,820 | 1,398 |
|  | Constante | -2,990 | ,745 | 16,103 | 1 | ,000 | ,050 |  |  |
| a. Variable(s) entrées à l'étape 1 : Fuite_LCR_01, Duree_KT_tot, Duree_KT_tot * Fuite_LCR_01 . | | | | | | | | | |

***Model with 2 way interaction term between LOC and High pathogen presence at the skin site***

| **Variables dans l'équation** | | | | | | | | | |
| --- | --- | --- | --- | --- | --- | --- | --- | --- | --- |
|  | | A | E.S. | Wald | ddl | Sig. | Exp(B) | IC pour Exp(B) 95% | |
|  |  |  |  |  |  |  |  | Inférieur | Supérieur |
| Etape 1^a^ | Duree_KT_tot | ,019 | ,046 | ,164 | 1 | ,686 | 1,019 | ,931 | 1,115 |
|  | High_pathogen_peau(1) | ,946 | 2,213 | ,183 | 1 | ,669 | 2,575 | ,034 | 196,925 |
|  | Duree_KT_tot by High_pathogen_peau(1) | ,084 | ,127 | ,439 | 1 | ,508 | 1,088 | ,848 | 1,395 |
|  | Constante | -2,354 | ,744 | 10,001 | 1 | ,002 | ,095 |  |  |
| a. Variable(s) entrées à l'étape 1 : Duree_KT_tot, High_pathogen_peau, Duree_KT_tot * High_pathogen_peau . | | | | | | | | | |

***Model with 2 way interaction term between High pathogen presence at the skin site and CSF leak***

| **Variables dans l'équation** | | | | | | | | | |
| --- | --- | --- | --- | --- | --- | --- | --- | --- | --- |
|  | | A | E.S. | Wald | ddl | Sig. | Exp(B) | IC pour Exp(B) 95% | |
|  |  |  |  |  |  |  |  | Inférieur | Supérieur |
| Etape 1^a^ | Fuite_LCR_01(1) | 2,721 | ,783 | 12,078 | 1 | ,001 | 15,200 | 3,276 | 70,530 |
|  | High_pathogen_peau(1) | 3,009 | ,892 | 11,367 | 1 | ,001 | 20,267 | 3,525 | 116,533 |
|  | Fuite_LCR_01(1) by High_pathogen_peau(1) | -2,316 | 1,642 | 1,989 | 1 | ,158 | ,099 | ,004 | 2,466 |
|  | Constante | -2,721 | ,462 | 34,742 | 1 | ,000 | ,066 |  |  |
| a. Variable(s) entrées à l'étape 1 : Fuite_LCR_01, High_pathogen_peau, Fuite_LCR_01 * High_pathogen_peau . | | | | | | | | | |
